# Supplementary material for: De novo discovery of conserved gene clusters in microbial genomes with Spacedust
Source: Nat Methods. 2025 Sep 15;22(10):2065–73. doi: 10.1038/s41592-025-02816-x (PMC12510874; doi:10.1038/s41592-025-02816-x)
Supplement: Supplementary file 1 — Supplementary Notes, Methods and Tables 1–3 [file 41592_2025_2816_MOESM1_ESM.pdf]

nature methods

Supplementary information https://doi.org/10.1038/s41592-025-02816-x

De novo discovery of conserved gene clusters in microbial genomes with Spacedust

In the format provided by the authors and unedited

CONTENTS

I. Supplementary Notes 1

A. Eukaryotic genomes 1

II. Supplementary Methods 2

A. Cluster definition 2

B. Cluster detection (step 5 in Fig. 1) 2

1. Clustering P-value 2

2. Ordering P-value. 3

3. Multihit P-value 5

C. Greedy hierarchical agglomerative clustering algorithm (step 6 in Fig. 1) 6

1. Gene fusion 7

III. Computational resources 8

IV. Software versions 9

References 10

V. Supplementary Tables 11

I. SUPPLEMENTARY NOTES

A. Eukaryotic genomes

Statistically significant gene clustering has been observed primarily in metabolic gene clusters in fungi and plants [1], and it is reported in multiple other taxa including humans [2]. Spacedust currently does not support input extraction of gene sequences with exon/intron structure from genomic sequences. However, as eukaryotes generally lack operons, these clusters possess different properties in organization and conservation patterns, such as overall much weaker clustering and clustering of unrelated functional categories [3, 4]. Therefore, parameters need to be carefully chosen according to prior knowledge. By leveraging its ability to detect remote homology, Spacedust could contribute to systematically identifying gene clusters conserved across different kingdoms [1]. This is future work.

II. SUPPLEMENTARY METHODS

A. Cluster definition

In Spacedust, a conserved gene cluster is defined as a group of neighboring genes on a query genome that has matched proteins that are positionally clustered in the target genome, with at least partial conservation of gene neighborhood. Here, the member genes of the cluster need not be strictly in the same order and direction in another genome, and a user-defined number of gaps (non-cluster genes) are allowed between any pairs of members.

B. Cluster detection (step 5 in Fig. 1)

Suppose we obtain a list of K pairwise best hits between a query proteome of size N_Q and a target proteome of size N_T. So for each $l \in \{1, \ldots, K\}$, we know the position $i_l \in \{1, \ldots, N_\mathrm{Q}\}$ of the query sequence in the query genome and the position $j_l \in \{1, \ldots, N_\mathrm{T}\}$ of the matched target sequence in the target genome.

Our goal is to find clusters of hits $\mathcal{C}$ from K hits such that the hits cluster closely together and have a partially conserved strand and order in both query and target genome. In these cases, the clusters are likely to be part of two groups of functionally associated, positionally orthologous proteins. We assess the degree of conservation of gene neighborhood of any cluster ($\mathcal{C}$) based on two P-value statistics, a clustering P-value $p_{clu}(\mathcal{C})$ and an ordering P-value $p_{ord}(\mathcal{C})$. Both statistics are independent of each other and of individual pairwise sequence similarity, and thus should improve the search sensitivity.

1. *Clustering P-value*

We want to assess the degree of clustering of the hits by computing a clustering P-value $p_\mathrm{clu}(\mathcal{C})$. Given any cluster of hits $\mathcal{C} \subset \{1, \ldots, K\}$ that contains $k = |\mathcal{C}|$ hits, we can define its span m as the maximum distance of the cluster members measured as the difference of gene position index on query and target genome:

$$m = \mathrm{span}(\mathcal{C}) = \max\{i_\mathrm{max}(\mathcal{C}) - i_\mathrm{min}(\mathcal{C}), j_\mathrm{max}(\mathcal{C}) - j_\mathrm{min}(\mathcal{C})\}\,. \tag{1}$$

where

$$
\begin{aligned}
i_{\min}(\mathcal{C}) &= \min\{i_l : l \in \mathcal{C}\},\\
i_{\max}(\mathcal{C}) &= \max\{i_l : l \in \mathcal{C}\},\\
j_{\min}(\mathcal{C}) &= \min\{j_l : l \in \mathcal{C}\},\\
j_{\max}(\mathcal{C}) &= \max\{j_l : l \in \mathcal{C}\}.
\end{aligned}
\tag{2}
$$

The P-value for this statistic is the probability of observing at least k hits in a square of positions with a span of m, where each position has a probability of q_0 (default 10^{-3}) of being hit. There are $\binom{m}{k}$ configurations to select k matched proteins from among the m query proteins and the same number to select k matched target proteins from among m. There are $k!$ ways to match the k queries to the k targets. The clustering P-value is the probability for a square of $m \times m$ positions in the match matrix to contain k or more matches, where the probability for a cell in this matrix to contain a match is $q_0 \ll 1$. Therefore, the P-value is

$$
p_{\text{clu}}(\mathcal{C}) = \sum_{k'=k}^{m} \binom{m}{k'}^2 k'!\, q_0^{k'} = \sum_{k'=k}^{m} \frac{m!^2}{(m-k')!^2 k'!} q_0^{k} \approx \frac{m!^2}{(m-k)!^2 k!} q_0^{k}. \tag{3}
$$

In the last step, we have approximated the sum with its first term since the second term is smaller than the first by a factor $q_0(m-k)^2/(k+1) \ll 1$, and similarly all other terms are negligible in comparison to the previous one.

The clustering P-value $p_{\text{clu}}(\mathcal{C})$ is independent of the size of query N_Q and target genome N_T, which can be applied to fragmented contigs containing few genes. The user can adjust the value of q_0 for the stringency of the clustering.

2. Ordering P-value.

For clusters of hits $\mathcal{C} \subset \{1, \ldots, K\}$ we want to assess the degree of conservation of their order and transcription directionality (strand) by computing an ordering P-value $p_{\text{ord}}(\mathcal{C})$. For any given cluster $\mathcal{C}$ of k hits, we re-index the hits as $(i_l, j_l), l \in \{1, \ldots, k\}$, with position index in the query set i_l sorted in ascending order, $i_1 < i_2 < \ldots < i_k$. We also denote the direction of transcription (strand of the encoding ORF) as $d_l \in \{-1, +1\}$ (+1 for forward strand, -1 for reverse strand) for the l'th query protein and $d'_l \in \{-1, +1\}$ for the corresponding, matched target protein.

We define the directionality and ordering statistic as the number of query protein pairs (i_l, i_{l+1}) whose matched target proteins (j_l, j_{l+1}) have the same direction and order.

$$n = \sum_{l=1}^{k-1} I\left(d_l\, d'_l = \text{sign}(j_{l+1} - j_l)\right)\, I\left(d_{l+1}\, d'_{l+1} = \text{sign}(j_{l+1} - j_l)\right)\,, \tag{4}$$

where $I(\cdot)$ is the indicator function. The expression in the summation is equal to 1 only in two cases: (1) the target protein (gene) pair have the same order as in the query, $j_{l+1} - j_l > 0$, and the direction of each hit are the same, d_{l+1} and d'_{l+1} have the same sign and d_l and d'_l have the same sign, (2) the target protein(gene) pair have the reversed order as in the query, $j_{l+1} - j_l < 0$, and d_l and d'_l have opposite signs. The second case occurs when the genes are encoded on opposite strands in the query and target genomes, i.e., $(+, -)$ or $(-, +)$.

The ordering P-value is equal to the number of configurations under n constraints for $k-1$ pairs divided by the total number of possible configurations. For a cluster of k hits, there are $2^k\, k!$ total possible combinations of ordering and direction. But since the choice of forward strand in the target genome is arbitrary, we only need to consider half of the configurations, $C(k) = 2^{k-1}\, k!$. For a conserved pair of hits (i_l, i_{l+1}) the constraint determines the sign of $j_{l+1} - j_l$ and of $d_{l+1} d'_{l+1}$ given the sign of $d_l d'_l$. Therefore, each pair with such a constraint behaves in a combinatorial sense as a single transcription unit, and therefore each such constraint reduces the effective number of independent transcription units by 1. According to equation (4) the number of these constraints is n. There are $k - 1$ pairs which could be constrained in such a way, hence there are $\binom{k-1}{n}$ configurations in which at least n pairs are constrained. So the number of configurations under n or more constraints is therefore $C(k - n)\binom{k-1}{n}$.

The ordering P-value is the number of configurations under at least n constraints $C(k - n)\binom{k-1}{n}$ divided by the total number $C(k) = 2^{k-1}\, k!$ of possible configurations :

$$p_{\text{ord}}(\mathcal{C}) = \frac{C(k-n)}{C(k)} \binom{k-1}{n} = \frac{2^{k-n-1}(k-n)!}{2^{k-1}\, k!} \frac{(k-1)!}{(k-n-1)!\, n!} \tag{5}$$

or

$$p_{\text{ord}}(\mathcal{C}) = \frac{1 - n/k}{2^n\, n!}\,. \tag{6}$$

3. Multihit P-value

We consider a cluster composed of k pairwise hit with best-hit P-values $p_1, \ldots, p_k$. In order to provide a summary statistic of the homologous relationship of a cluster, we need to take into account the strength and the number of individual hits. We assume that if a sizable fraction of the hits have a significant similarity, it already constitutes a signal for a global significant similarity. A standard approach is to use the Fisher's method [5] for combining k independent P-values of the individual hits that are the product of all P-values:

$$F = \prod_{i=1}^{k} p_i \tag{7}$$

However, the Fisher's method is not suitable when many P-values in the cluster are not significant, as they will dilute the actual significant homology signals. In order to tackle the issue, the previous works introduced a truncated version of the Fisher's method that takes into account only P-values below a given p_0 threshold [7]. As a drawback, the truncated F statistic has a strong discontinuity when a single P-value falls below the threshold. We recall that each or the pairwise hits between sequences in the query and target sets has to have a best-hit P-value less than or equal to some threshold $p_0 = 1/(N_{\mathrm{T}} + 1)$. We consider a cluster composed of k pairwise hit with P-values $p_1, \ldots, p_k < p_0$. We propose a variant R of the truncated F statistic, that allows a continuous increase of the R-value by summing up the parts of evidence that pass the threshold p_0:

$$R = -\sum_{i=1}^{k} \log \frac{p_i}{p_0} \tag{8}$$

Because the P-values of pairwise hits are uniformly distributed over $[0, 1]$ under the null model, the subset of P-values smaller than p_0 are also uniformly distributed, and the p_i/p_0 are therefore uniformly distributed on $[0, 1]$. The multi-hit R statistic can distinguish homology signals from random cases, while still reflecting the strength and number of individual hits. The P-value for a variable equal to the product of these k independent P-values is well

known [5, 6]:

$$\begin{aligned} \text{P-value}(e^{-r}) &= p\left(\prod_{i=1}^{k}\frac{p_i}{p_0}\leq e^{-r}\middle| p_1,\ldots,p_k\sim U(0,p_0)\right) \\ &= p\left(\prod_{i=1}^{k}p'_i\leq e^{-r}\middle| p'_1,\ldots,p'_k\sim U(0,1)\right) \\ &= e^{-r}\sum_{i=0}^{k-1}\frac{r^i}{i!}\,, \end{aligned} \tag{9}$$

where r follows the R statistic.

This multihit P-value is distributed according to $U(0,1)$ under the null model. It has some similarity with the standard E-value used for pairwise matches in sequence searching: It already takes account of the size of the target set (by dint of the cut-off p_0 on the pairwise hit P-values), and it does not correct for multiple testing with respect to the multiple queries.

C. Greedy hierarchical agglomerative clustering algorithm (step 6 in Fig. 1)

This algorithm is used for detecting cluster of hits with conserved gene neighborhood. We obtain the clustering and ordering P-values $p_{clu}(\mathcal{C})$ and $p_{ord}(\mathcal{C})$ separately for any cluster of hits $\mathcal{C}$, of which the calculation is detailed in the subsections II B 1 and II B 2. These two P-values are independent random variables under the null model. We can therefore combine them using the product of P-values[6]

$$p := p_{\text{clu}}(\mathcal{C})\, p_{\text{ord}}(\mathcal{C}). \tag{10}$$

The P-value of p, termed cluster match P-value, is

$$f(p) = p(1 - \log p). \tag{11}$$

We can define a cluster score $S(\mathcal{C})$ as the minus natural logarithm of the cluster match P-value $f(p)$:

$$S(\mathcal{C}) := -\log f(p) = -\log p_{\text{clu}}(\mathcal{C}) - \log p_{\text{ord}}(\mathcal{C}) + \log\left(1 - \log p_{\text{clu}}(\mathcal{C}) - \log p_{\text{ord}}(\mathcal{C})\right). \tag{12}$$

We propose a greedy algorithm for identifying cluster $\mathcal{C}$ of pairwise hits with a maximum score $S(\mathcal{C})$, since finding the cluster of minimum P-value is equivalent to maximizing the

score $S(\mathcal{C})$. Due to the monotonicity of $f(p)$, we only need to maximize the first part of the equation $-\log p_{\mathrm{clu}}(\mathcal{C}) - \log p_{\mathrm{ord}}(\mathcal{C})$, which is faster to compute in practice.

The agglomerative hierarchical clustering algorithms treat each hit as a singleton cluster in the beginning and then successively merge pairs of clusters until all clusters have been merged into a single cluster that contains all hits [8]. The algorithm proceeds as follows:

1. Add each hit l as a singleton cluster to the list of clusters $\mathcal{L}$.

2. Compute the scores $S(\mathcal{C} \cup \mathcal{C}')$ using equation (12) for all pairs of *compatible* clusters $\mathcal{C}, \mathcal{C}' \in \mathcal{L}$, where compatibility requires that at most $d-1$ genes lie between the genes or the two clusters in the query genome and likewise in the target genome,

$$\begin{aligned} &\min\{|j_{\min}(\mathcal{C}) - j_{\max}(\mathcal{C}')|\,,\, |j_{\min}(\mathcal{C}') - j_{\max}(\mathcal{C})|\} \le d \text{ and} \\ &\min\{|i_{\min}(\mathcal{C}) - i_{\max}(\mathcal{C}')|\,,\, |i_{\min}(\mathcal{C}') - i_{\max}(\mathcal{C})|\} \le d \end{aligned} \tag{13}$$

and no protein in the query set or target set must be matched more than once, i.e $\forall\, l \in \mathcal{C}, l' \ \in \mathcal{C}' : i_l \neq i_{l'} \wedge j_l \neq j_{l'}$.

3. While the largest score $S(\mathcal{C} \cup \mathcal{C}')$ for merging compatible clusters exceeds some merging threshold S_{merge},

4. remove $\mathcal{C}$ and $\mathcal{C}'$ from $\mathcal{L}$ and all their merge scores with other clusters from the score list;

5. add $\mathcal{C} \cup \mathcal{C}'$ to $\mathcal{L}$ and add the merge scores of $\mathcal{C} \cup \mathcal{C}'$ with all compatible clusters in $\mathcal{L}$ to the score list.

6. Return all non-singleton clusters in $\mathcal{L}$ whose score is above a significance threshold $S_{\min}$.

The merging threshold S_{merge} is derived from d as the maximum cluster score of a cluster of two elements with a span of $d-1$. The maximum number of genes allowed between two clusters d and the significance threshold S_{min} for reporting the clusters are both user-definable parameters, where S_{min} is converted to cluster match P-value as in equation (11). Simultaneously, the reported cluster has to satisfy a user-defined multihit P-value threshold, of which the computation is detailed in subsection II B 3.

1. Gene fusion

In the agglomerate clustering step, we do not allow any query or target protein to be matched more than once. Comparative genomic revealed cases in which two or more proteins encoded separately in one genome appear as fusions either in the same genome or some

other genomes. Such fusion proteins, termed Rosetta stone sequences, are also strongly suggestive of functional relationships of the disparate proteins, and thus should be detected and reported in the cluster output [9].

On the sequence level, a gene fusion case has to qualify for the following criteria: (1) at least two hits match to the same target protein (2) the hits do not overlap or only overlaps very little on the target protein (3) a significant part of the target protein is covered by the query proteins (4) a significant part of the query proteins are covered by the target protein and (5) the order and direction of transcription is retained. In the homology search step, we demand a query coverage of ≥80%. Only one best hit in target set is chosen for each query protein.

To include gene fusion events, we allow two proteins encoded by consecutive genes at position i_l, i_{l+1} in query set to be matched to one protein $j_{l'}$ in the target, only if the two aligned regions in the one protein do not overlap, and the aligned region has to cover ≥80% of the target protein, and the order and direction of transcription are conserved.

III. COMPUTATIONAL RESOURCES

All runs are performed on the GWDG HPC cluster (www.gwdg.de) using individual nodes with two Intel Xeon E5-2640v3 processors at 2.6 GHz each possessing 2 × 64 cores and 1024 GB RAM.

IV. SOFTWARE VERSIONS

Software	Version
Spacedust	Git: 2-e56c505
Foldseek	10-941cd33
ProstT5	v0.0.1
Prodigal	2.6.3
eggNOG-mapper	v2.0
Mash	v2.3
PADLOC	v1.1.0
ClusterFinder	Git: 5ee2c15
DeepBGC	v0.1.29
AntiSMASH	v8.0.0
GECCO	v0.9.10

Benchmark data analysis and visualization was done with:

Software/Package	Version
R	4.1.2
ggplot2	3.4.2
cowplot	1.1.1
gggenes	0.5.5
python	3.10.2
matplotlib	3.5.1
ScyPy	1.8.0

[1] Foflonker, F. & Blaby-Haas, C. Colocality to Cofunctionality: Eukaryotic Gene Neighborhoods as a Resource for Function Discovery. *Molecular Biology And Evolution.* **38**, 650-662 (2021).

[2] Lee, J. & Sonnhammer, E. Genomic gene clustering analysis of pathways in eukaryotes. *Genome Research.* **13**, 875-882 (2003).

[3] Nützmann, H., Scazzocchio, C. & Osbourn, A. Metabolic Gene Clusters in Eukaryotes. *Annual Review Of Genetics.* **52**, 159-183 (2018).

[4] Mihelčić, M., Šmuc, T. & Supek, F. Patterns of diverse gene functions in genomic neighborhoods predict gene function and phenotype. *Scientific Reports.* **9**, 19537 (2019).

[5] Fisher, R. A., N. Statistical methods for research workers (4th ed.). *Oliver & Boyd.* . **17**, 48 (1932).

[6] Bailey, T. & Grundy, W. Classifying proteins by family using the product of correlated p-values. *Proceedings Of The Third Annual International Conference On Computational Molecular Biology.* pp. 10-14 (1999)

[7] Zaykin, D., Zhivotovsky, L., Westfall, P. & Weir, B. Truncated product method for combining P-values. *Genetic Epidemiology.* **22**, 170-185 (2002,2)

[8] Everitt, B., Landau, S., Leese, M. & Stahl, D. Cluster Analysis. (Wiley,2010)

[9] Date, S. The Rosetta Stone Method. *Bioinformatics: Structure, Function And Applications.* pp. 169-180 (2008)

V. SUPPLEMENTARY TABLES

Supplementary Table I. EggNOG-mapper annotation of Synechocystis sp. PCC6803 clusters

Index	Start	End	COG category	Name	EC	KEGG_ko	KEGG_Module	PFAMs
510	569141	569488	C	rub	-	-	-	Rubredoxin
511	569544	570572	S	ycf48	-	-	-	PSII_BNR
512	570658	570903	C	psbE	-	ko:K02707	M00161	Cytochrom_B559,Cytochrom_B559a
513	570940	571074	A	psbF	-	ko:K02708	M00161	Cytochrom_B559
514	571084	571203	U	psbL	-	ko:K02713	-	PsbL
515	571236	571355	U	psbJ	-	ko:K02711	-	PsbJ
528	583092	582481	S	-	-	-	-	Uma2
529	583573	583157	P	-	-	ko:K03711	-	FUR
530	583680	584696	P	zntC	-	ko:K09815	M00242	ZnuA
531	584674	585543	P	-	-	ko:K09817	M00242	ABC_tran
532	585497	586342	U	-	-	ko:K09816	M00242	ABC-3
542	600833	599910	J	dnaJ3	-	ko:K05516	-	DnaJ,DnaJ_C
543	603308	600942	O	dnaK1	-	ko:K04043	-	HSP70
583	644379	643549	S	-	3.1.2.12	ko:K01070	-	Esterase
584	645497	644388	C	frmA	1.1.1.1,1.1.1.284	ko:K00121	-	ADH_N,ADH_zinc_N
586	658262	659335	H	chlI	6.6.1.1	ko:K03405	-	Mg_chelatase
587	659370	660584	J	tyrS	6.1.1.1	ko:K01866	M00359,M00360	S4,tRNA-synt_1b
590	662952	663188	J	tyrS	6.1.1.1	ko:K01866	M00359,M00360	S4,tRNA-synt_1b
591	663342	663731	L	ycf41	-	ko:K03111	-	SSB
605	673889	675340	C	cydA	1.10.3.14	ko:K00425	M00153	Cyt_bd_oxida_I
606	675344	676354	C	cydB	1.10.3.14	ko:K00426	M00153	Cyt_bd_oxida_II
608	678286	677045	F	ackA	2.7.2.1	ko:K00925	M00357,M00579	Acetate_kinase
609	679023	678283	Q	clcD	3.1.1.45	ko:K01061	-	DLH
616	684156	683140	P	pobA	-	-	-	Rieske
617	684439	684143	C	-	-	-	-	Fer2
618	687334	684560	NT	-	-	ko:K02487	M00507	CheW,H-kinase_dim,HATPase_c,Hpt,Response_reg
619	690252	687391	NT	pilJ	-	ko:K02660	-	GAF,HAMP,MCPsignal
620	690866	690399	NT	-	-	ko:K02659	-	CheW
621	691264	690899	KT	-	-	ko:K02658	M00507	Response_reg
622	692576	691371	KT	-	-	ko:K02657	M00507	DUF4388,Response_reg
624	695874	695290	O	-	-	-	-	AhpC-TSA
627	698723	698962	P	-	-	ko:K04758	-	FeoA
628	698970	700814	P	feoB	-	ko:K04759	-	FeoB_C,FeoB_N,Gate
631	704390	704845	S	-	-	ko:K09775	-	DUF212

Supplementary Table II. EggNOG-mapper annotation of Synechocystis sp. PCC6803 clusters (continued)

Index	Start	End	COG category	Name	EC	KEGG_ko	KEGG_Module	PFAMs
648	724345	724094	P	cpcD	-	ko:K02287	-	CpcD
649	725367	724492	H	cpcC	-	ko:K02286	-	CpcD,PBS_linker_poly
650	726234	725413	H	cpcC2	-	ko:K02286	-	CpcD,PBS_linker_poly
651	726837	726349	C	cpcA	-	ko:K02284	-	Phycobilisome
652	727466	726948	C	cpcB	-	ko:K02285	-	Phycobilisome
655	731358	730108	KLT	spkA	2.7.11.1	ko:K12132	-	Pkinase
656	731674	731363	KLT	spkA	2.7.11.1	ko:K12132	-	Pkinase
657	732062	731748	-	-	-	-	-	-
671	747234	747578	P	-	-	ko:K05567	-	Oxidored_q2
672	747575	749038	CP	ndhD5	-	ko:K05568	-	Proton_antipo_M
673	749150	749500	P	-	-	ko:K05567	-	Oxidored_q2
674	749550	751037	CP	ndhD5	-	ko:K05568	-	Proton_antipo_M
675	751034	751426	P	-	-	ko:K05569	-	MNHE
676	751381	751674	-	-	-	ko:K05570	-	-
677	751671	751967	P	-	-	ko:K05571	-	PhaG_MnhG_YufB
678	751936	752535	P	-	-	ko:K07242	-	DUF4040
679	752532	753179	P	-	-	ko:K05566	-	MnhB
682	756053	757195	NU	-	-	-	-	N_methyl
683	757244	757720	NU	-	-	-	-	N_methyl
684	757671	758753	NU	-	-	-	-	N_methyl
685	758829	761228	NU	-	-	-	-	-
708	790719	791717	C	coxB	1.9.3.1	ko:K02275	M00155	COX2,COX2_TM
709	791806	793461	C	ctaDI	1.9.3.1	ko:K02274	M00155	COX1
710	793509	794210	C	ctaE	1.9.3.1	ko:K02276	M00155	COX3
713	796732	795932	P	tauB	-	ko:K02049	M00188	ABC_tran
714	797631	796789	P	-	-	ko:K02050	M00188	BPD_transp_1
715	798792	797734	P	-	-	ko:K02051	M00188	NMT1,NMT1_2
716	799535	798846	KO	hypB	-	ko:K04652	-	cobW
717	799958	799596	S	hypA	-	ko:K04651	-	HypA
718	801206	800034	E	speB2	3.5.3.11	ko:K01480	M00133	Arginase
719	801643	802152	T	-	-	-	-	GAF
720	802207	803238	T	-	-	-	-	GAF,GGDEF
730	819979	817967	G	tktA	2.2.1.1	ko:K00615	M00004,M00007,M00165,M00167	Transket_pyr,Transketolase_C,Transketolase_N
731	821352	820102	I	fabF	2.3.1.179	ko:K09458	M00083,M00572	Ketoacyl-synt_C,ketoacyl-synt
732	821783	821550	IQ	acpP	-	ko:K02078	-	PP-binding

Supplementary Table III. EggNOG-mapper annotation of Synechocystis sp. PCC6803 clusters (continued)

Index	Start	End	COG category	Name	EC	KEGG_ko	KEGG_Module	PFAMs
740	826882	826637	J	rpmE	-	ko:K02909	M00178	Ribosomal_L31
741	827348	826935	J	rps9	-	ko:K02996	M00178,M00179	Ribosomal_S9
742	827803	827348	J	rplM	-	ko:K02871	M00178,M00179	Ribosomal_L13
743	828695	827868	J	truA	5.4.99.12	ko:K06173	-	PseudoU_synth_1
744	829063	828713	J	rplQ	-	ko:K02879	M00178	Ribosomal_L17
745	830085	829141	K	rpoA	2.7.7.6	ko:K03040	M00183	RNA_pol_A_CTD,RNA_pol_A_bac,RNA_pol_L
746	830586	830194	J	rpsK	-	ko:K02948	M00178,M00179	Ribosomal_S11
747	831018	830635	J	rpsM	-	ko:K02952	M00178,M00179	Ribosomal_S13
748	831217	831101	J	rpmJ	-	ko:K02919	M00178	Ribosomal_L36
749	831558	831355	J	infA	-	ko:K02518	-	eIF-1a
750	832265	831702	F	adk	2.7.4.3	ko:K00939	M00049	ADK
751	833719	832391	U	secY	-	ko:K03076	M00335	SecY
752	834264	833821	J	rplO	-	ko:K02876	M00178,M00179	Ribosomal_L27A
753	834854	834333	J	rps5	-	ko:K02988	M00178,M00179	Ribosomal_S5,Ribosomal_S5_C
754	835256	834894	J	rplR	-	ko:K02881	M00178,M00179	Ribosomal_L18p
755	835799	835260	J	rpl6	-	ko:K02933	M00178,M00179	Ribosomal_L6
756	836260	835859	J	rps8	-	ko:K02994	M00178,M00179	Ribosomal_S8
757	836954	836352	J	rpl5	-	ko:K02931	M00178,M00179	Ribosomal_L5,Ribosomal_L5_C
758	837348	837001	J	rplX	-	ko:K02895	M00178,M00179	KOW,ribosomal_L24
759	837717	837349	J	rplN	-	ko:K02874	M00178,M00179	Ribosomal_L14
760	837986	837741	J	rpsQ	-	ko:K02961	M00178,M00179	Ribosomal_S17
761	838215	837994	J	rpmC	-	ko:K02904	M00178,M00179	Ribosomal_L29
762	838637	838218	J	rplP	-	ko:K02878	M00178	Ribosomal_L16
763	839400	838678	J	rps3	-	ko:K02982	M00178,M00179	KH_2,Ribosomal_S3_C
764	839795	839430	J	rplV	-	ko:K02890	M00178,M00179	Ribosomal_L22
765	840089	839811	J	rpsS	-	ko:K02965	M00178,M00179	Ribosomal_S19
766	840952	840122	J	rpl2	-	ko:K02886	M00178,M00179	Ribosomal_L2,Ribosomal_L2_C
767	841307	841002	J	rplW	-	ko:K02892	M00178,M00179	Ribosomal_L23
768	841932	841300	J	rpl4	-	ko:K02926	M00178	Ribosomal_L4
769	842613	841972	J	rpl3	-	ko:K02906	M00178,M00179	Ribosomal_L3
782	852898	852470	L	-	-	ko:K07492	-	DDE_Tnp_1_2
783	853281	852895	L	-	-	ko:K07492	-	DUF4096
784	857450	853497	K	rpoC2	2.7.7.6	ko:K03046	M00183	RNA_pol_Rpb1_3,RNA_pol_Rpb1_4,RNA_pol_Rpb1_5
785	860866	857558	K	rpoB	2.7.7.6	ko:K03043	M00183	RNA_pol_Rpb2_1,RNA_pol_Rpb2_2,RNA_pol_Rpb2_3,RNA_pol_Rpb2_45,R
786	861737	860952	L	dtd3	-	ko:K03424	-	TatD_DNase
787	862166	861873	J	rpsT	-	ko:K02968	M00178	Ribosomal_S20p
