# Supplementary material for: De novo discovery of conserved gene clusters in microbial genomes with Spacedust
Source: Nat Methods. 2025 Sep 15;22(10):2065–73. doi: 10.1038/s41592-025-02816-x (PMC12510874; doi:10.1038/s41592-025-02816-x)
Supplement: Supplementary file 2 — Reporting Summary [file 41592_2025_2816_MOESM2_ESM.pdf]

nature portfolio

Corresponding author(s): Johannes Soeding

Last updated by author(s): Jul 22, 2025

Reporting Summary

Nature Portfolio wishes to improve the reproducibility of the work that we publish. This form provides structure for consistency and transparency in reporting. For further information on Nature Portfolio policies, see our Editorial Policies and the Editorial Policy Checklist.

Statistics

For all statistical analyses, confirm that the following items are present in the figure legend, table legend, main text, or Methods section.

n/a	Confirmed	
☐	☒	The exact sample size (*n*) for each experimental group/condition, given as a discrete number and unit of measurement
☒	☐	A statement on whether measurements were taken from distinct samples or whether the same sample was measured repeatedly
☒	☐	The statistical test(s) used AND whether they are one- or two-sided<br>*Only common tests should be described solely by name; describe more complex techniques in the Methods section.*
☒	☐	A description of all covariates tested
☒	☐	A description of any assumptions or corrections, such as tests of normality and adjustment for multiple comparisons
☒	☐	A full description of the statistical parameters including central tendency (e.g. means) or other basic estimates (e.g. regression coefficient) AND variation (e.g. standard deviation) or associated estimates of uncertainty (e.g. confidence intervals)
☐	☒	For null hypothesis testing, the test statistic (e.g. *F*, *t*, *r*) with confidence intervals, effect sizes, degrees of freedom and *P* value noted<br>*Give P values as exact values whenever suitable.*
☒	☐	For Bayesian analysis, information on the choice of priors and Markov chain Monte Carlo settings
☒	☐	For hierarchical and complex designs, identification of the appropriate level for tests and full reporting of outcomes
☒	☐	Estimates of effect sizes (e.g. Cohen's *d*, Pearson's *r*), indicating how they were calculated

Our web collection on statistics for biologists contains articles on many of the points above.

Software and code

Policy information about availability of computer code

Data collection

Spacedust is free open-source (GPLv3) software at: https://github.com/soedinglab/spacedust.

Software used as part of Spacedust:
MMseqs2 (15-6f452) :https://mmseqs.com/
Foldseek (10-941cd33): https://github.com/steineggerlab/foldseek
ProstT5 (v0.0.1):https://github.com/mheinzinger/ProstT5

Software used for annotating genes/proteins:
Prodigal (2.6.3): https://github.com/hyattpd/Prodigal
eggNOG-mapper (v2.0): https://github.com/eggnogdb/eggnog-mapper
AntiSMASH (v6.0.0): https://github.com/antismash/antismash

Software used for selection of genomes:
Mash (v2.3): https://github.com/marbl/Mash

Benchmarked software:
PADLOC (v1.1.0): https://github.com/padlocbio/padloc
ClusterFinder (Git: 5ee2c15): https://github.com/petercim/ClusterFinder
DeepBGC (v0.1.29): https://github.com/Merck/deepbgc
GECCO(v0.9.10):https://github.com/zellerlab/GECCO

Data analysis	Benchmark data and visualization was done with R/4.1.2, ggplot2/3.4.2, cowplot 1.1.1, gggenes/0.5.5, python/3.10.2, matplotlib/3.5.1 SciPy/1.8.0.<br>Scripts and data for data analysis are deposited in: https://wwwuser.gwdg.de/~compbiol/spacedust/

For manuscripts utilizing custom algorithms or software that are central to the research but not yet described in published literature, software must be made available to editors and reviewers. We strongly encourage code deposition in a community repository (e.g. GitHub). See the Nature Portfolio guidelines for submitting code & software for further information.

Data

Policy information about availability of data

All manuscripts must include a data availability statement. This statement should provide the following information, where applicable:

- Accession codes, unique identifiers, or web links for publicly available datasets
- A description of any restrictions on data availability
- For clinical datasets or third party data, please ensure that the statement adheres to our policy

Data used in this work were obtained from the public sources and are freely accessible. Bacterial genome dataset was assembled from the KEGG GENOME collection (https://www.genome.jp/kegg/tables/br08606.html) downloaded from NCBI GenBank in 09/2022. The protein structure database used for mapping was compiled from the AlphaFold DB (https://alphafold.ebi.ac.uk/). The genomes and datasets used for the analysis in Fig. 5 and Supplementary Figs. 7–9 are publicly accessible via the supplementary material of Hannigan, G.D. et al. (https://doi.org/10.1093/nar/gkz654). The genome used as a query in Fig. 6 is available from NCBI GenBank under accession number NZ_BEXT01000001.1 (https://www.ncbi.nlm.nih.gov/nuccore/BEXT01000001.1). The target database is publicly available from GTDB (https://gtdb.ecogenomic.org/).
There are no restrictions on data availability, and all materials can be accessed freely.

Human research participants

Policy information about studies involving human research participants and Sex and Gender in Research.

Reporting on sex and gender	Not applicable
Population characteristics	Not applicable
Recruitment	Not applicable
Ethics oversight	Not applicable

Note that full information on the approval of the study protocol must also be provided in the manuscript.

Field-specific reporting

Please select the one below that is the best fit for your research. If you are not sure, read the appropriate sections before making your selection.

☒ Life sciences ☐ Behavioural & social sciences ☐ Ecological, evolutionary & environmental sciences

For a reference copy of the document with all sections, see nature.com/documents/nr-reporting-summary-flat.pdf

Life sciences study design

All studies must disclose on these points even when the disclosure is negative.

Sample size	Not applicable. Spacedust is an exclusively computational method and did not involve any experimental samples that would require sample size estimations, replications, blinding, or randomization.
Data exclusions	No data were excluded.
Replication	Not applicable. Spacedust is an exclusively computational method. The computational method is deterministic when run on the same computer setup. Therefore, replication is not needed as the result would be identical for each replicate.
Randomization	Not applicable.
Blinding	Not applicable.

Reporting for specific materials, systems and methods

We require information from authors about some types of materials, experimental systems and methods used in many studies. Here, indicate whether each material, system or method listed is relevant to your study. If you are not sure if a list item applies to your research, read the appropriate section before selecting a response.

Materials & experimental systems

n/a	Involved in the study
☒	☐ Antibodies
☒	☐ Eukaryotic cell lines
☒	☐ Palaeontology and archaeology
☒	☐ Animals and other organisms
☒	☐ Clinical data
☒	☐ Dual use research of concern

Methods

n/a	Involved in the study
☒	☐ ChIP-seq
☒	☐ Flow cytometry
☒	☐ MRI-based neuroimaging
